# Hotspots and Trends in Nursing Interventions for Breast Cancer Patients Undergoing Radiotherapy: A Bibliometric Analysis

**DOI:** 10.3390/nursrep16070210

**Published:** 2026-06-23

**Authors:** Mengdie Hu, Yongxing Bao, Wei Zheng, Yan Wang, Jiawen Fu, Xuechun Wang, Miao Sun, Huiying Tao, Zhouguang Hui

**Affiliations:** 1 Department of VIP Medical Services, National Cancer Center/National Clinical Research Center for Cancer/Cancer Hospital, Chinese Academy of Medical Sciences and Peking Union Medical College, Beijing 100021, China; 2 Department of Thoracic Surgery, National Cancer Center/National Clinical Research Center for Cancer/Cancer Hospital, Chinese Academy of Medical Sciences and Peking Union Medical College, Beijing 100021, China

**Keywords:** breast cancer, nursing interventions, radiotherapy, bibliometric, research frontier

## Abstract

**Background:** Research on nursing interventions for breast cancer patients undergoing radiotherapy is increasing. However, comprehensive mapping and synthesis regarding the field’s overall knowledge structure and development remain limited. This study aims to utilize bibliometric methods to analyze the current status, research hotspots, and emerging trends in this field. **Methods:** We conducted a bibliometric analysis of 256 publications from the Web of Science Core Collection and PubMed. **Results:** Publication volume showed a notable increase after 2020 (16–25 articles per year). The United States leads in output (82 articles, 32.0%), followed by China (25 articles). At the institutional level, the University of California, San Francisco (10 articles) is the most productive, while George Washington University leads in total citations (1759). *Oncology Nursing Forum* is the leading journal both in publication volume (20 articles) and h-index (13). Twelve major research clusters were identified, primarily focusing on symptom management (specifically pain) and psychosocial support. Keyword burst analysis suggests that current frontiers have shifted from acute symptom control toward systematic management approaches and psychological symptom interventions. **Conclusions:** Based on the analysis of 256 publications and 12 research clusters, this study indicates that the focus of nursing research appears to be expanding from acute symptom control toward comprehensive case management and targeted psychological research. These findings may provide useful directions for future research and clinical practice, particularly regarding the integration of psychosocial care into nursing management.

## 1. Introduction

Breast cancer stands as the most prevalent malignant tumor among females globally. Its high incidence and mortality rates present substantial challenges to public health [1,2]. Radiotherapy (RT), as a vital element of the multidisciplinary treatment for breast cancer, assumes a crucial position in post-lumpectomy care and in the treatment of patients at high risk after mastectomy. It is extensively employed as an adjuvant treatment for the chest wall and regional lymphatics. This helps ensure adequate local and regional control, particularly after surgical staging or axillary clearance [3,4]. This adjuvant therapy for the chest and axilla significantly curtails local recurrence rates and enhances survival outcomes [5,6]. Nonetheless, while RT offers considerable clinical advantages, it is also linked to a series of adverse effects, such as radiation dermatitis, fatigue, lymphedema, and psychological distress [7,8,9]. These symptoms severely influence patients’ treatment tolerance, quality of life, and rehabilitation progress [10].

Compared to physicians, nurses maintain more continuous and direct contact with patients. Accordingly, nursing care plays a particularly important role in symptom management, treatment adherence, and quality-of-life support during radiotherapy [11,12]. Although nurses do not direct primary oncological decision-making or diagnose disease progression, their comprehensive care is essential for supporting patients’ overall recovery. Specifically, nursing care is pivotal in managing locoregional adverse effects. These responsibilities include the care of surgical wounds, radiation-induced dermatitis, and lymphedema [13,14]. Moreover, nurses provide indispensable psychosocial support throughout long-term cancer survivorship [15,16].

Research has shown that nursing interventions can significantly enhance patients’ adherence to skin care protocols. They may also reduce RT induced cutaneous toxicities while improving patients’ overall well-being and treatment experience [17,18]. Additionally, cognitive behavioral therapy for patients undergoing RT has shown positive effects in alleviating fatigue and enhancing quality of life. These findings underscore the indispensable role of nurses in providing psychological support and facilitating behavioral change [19]. Furthermore, studies have emphasized the critical role of nurses in identifying and managing radiotherapy-related side effects. This contribution supports a more comprehensive and personalized patient care within a multidisciplinary framework [20,21].

A considerable volume of original research and reviews has explored nursing care strategies for breast cancer patients undergoing RT [22,23,24,25]. However, most of the available evidence focuses primarily on the efficacy of specific interventions or on the clinical role of nurses in radiotherapy care. For example, a scoping review of nursing care for women with breast cancer receiving radiotherapy summarized educational and informational interventions. It further indicated that psychoeducational programs may alleviate stress and depression during treatment [26]. Similarly, a review on advanced breast cancer highlighted the crucial contribution of specialized nurses during the radiotherapy process [27]. Their roles include symptom management, prevention of adverse effects, patient education, and support for quality of life. Moreover, studies in other radiotherapy settings, such as lung and esophageal cancer, have reported positive effects of evidence-based nursing, rehabilitation nursing, and integrated medical–nursing models. These approaches can improve treatment adherence, self-efficacy, emotional well-being, and quality of life [27,28]. Nevertheless, these studies are mainly narrative reviews or clinical reports centered on specific interventions. They do not clearly define the broader intellectual framework, collaboration patterns, and evolving research themes in this field.

To map the broader intellectual framework, researchers have increasingly employed bibliometric approaches in oncology nursing. For instance, previous bibliometric studies have explored the general panorama of breast cancer nursing research from 2009 to 2018 [29]. More recent studies have examined nurse-led survivorship models and patient-reported outcome workflows in patients with early-stage breast cancer [30]. However, the existing literature still shows important limitations. Previous bibliometric analyses provide general overviews of oncology nursing or long-term post-treatment survivorship. In contrast, they do not specifically address the acute treatment phase, particularly radiotherapy. Therefore, rather than conducting a broad survey of oncology nursing, this study aims to systematically delineate the developmental trajectory, collaboration patterns, and evolving hotspots of nursing care for breast cancer patients undergoing RT.

In this study, we conducted a comprehensive bibliometric analysis of papers related to nursing interventions for breast cancer patients undergoing radiotherapy. We systematically analyzed publication trends. In addition, we identified the most productive countries, institutions, and researchers. We also determined major research hotspots and emerging directions. This study aims to inform future research and support the development of nursing practice in this area.

## 2. Materials and Methods

### 2.1. Data Collection

A systematic search was conducted in the Web of Science Core Collection (WOSCC) and PubMed using the following query strategy. For the WOSCC database search, the following query was executed in the “Topic” field: TS=((Nursing Interventions OR Nursing Care OR Nursing Strategies OR Nursing Practices OR Oncology Nursing) AND (Breast Cancer OR Breast Neoplasms OR Breast Carcinoma OR Mammary Carcinoma OR Breast Tumors OR Breast Tumours) AND (Radiotherapy OR Radiation Therapy OR Radiation Treatment)). No start date was specified, and the end date was set as 16 September 2025.

Regarding the PubMed database search, a search strategy integrating Medical Subject Headings (MeSH) terms and free-text keywords was adopted. The core search string was as follows: ((“Nursing Care”[Mesh] OR “Oncology Nursing”[Mesh] OR nursing intervention[tiab] OR nursing care[tiab] OR nursing strateg[tiab] OR nursing practice[tiab]) AND (“Breast Neoplasms”[Mesh] OR breast cancer[tiab] OR breast neoplasm[tiab] OR breast carcinoma[tiab] OR mammary carcinoma[tiab] OR breast tumor*[tiab]) AND (“Radiotherapy”[Mesh] OR radiotherapy[tiab] OR radiation therapy[tiab] OR radiation treatment[tiab])). No start date was specified, and the end date was 16 September 2025.

For data collection, records from the WOSCC were exported in the Plain Text File format, which included the “Full Record and Cited References”, and the results from PubMed were exported in the “PubMed” format. Subsequently, all retrieved data from both databases were imported into NoteExpress (version = 3.8.0.9492) software. Duplicate records were identified and removed by matching a combination of fields, including article titles, authors, and publication year. Finally, the remaining deduplicated records were exported in the RefWorks-CiteSpace format for subsequent analysis.

To guarantee the quality and consistency of the bibliometric analysis, the inclusion criteria were clearly defined as: (1) peer-reviewed original research articles (covering all study designs, such as experimental, observational, and qualitative studies) and review papers; (2) publications written in English. Conversely, other non-primary document types (such as meeting abstracts, editorials, letters) were strictly excluded. All records retrieved from the two databases were initially imported. To ensure the relevance of the included papers, the full text of the initially retrieved publications was screened to exclude studies that were not directly relevant to the research topic. The screening process was independently carried out by two researchers to ensure that the selected studies were strictly in line with the research topic. Disagreements regarding study eligibility were resolved through discussion, and unresolved cases were adjudicated by a third senior researcher until consensus was achieved.

### 2.2. Data Analysis

Bibliometric analysis is a rigorous quantitative method for evaluating scientific publications. It utilizes statistical techniques to assess the literature and provides a powerful approach for visualizing scientific advancement and identifying emerging trends within a specific research domain [31]. This method enables systematic analysis of publication patterns, citation networks, and co-occurring keywords. It also provides an objective assessment of scientific output and academic influence. In the present study, we utilized literature records as input files and conducted statistical analyses using the following methods.

CiteSpace (version = 6.4.R1) [32] was utilized for keyword clustering, timeline views of keywords, and detection of explosive keywords. The parameters of CiteSpace were configured as follows: time slicing (from 2002 to 2025, years per slice = 1), node types (keyword), selection criteria (g-index k = 25), and pruning methods (Minimum Spanning Tree and pruning sliced networks). In the clustering analysis generated by the CiteSpace software, the modularity Q value (Q) and weighted mean silhouette S value (S) are crucial metrics for evaluating the robustness of network clustering [33,34]. The Q value measures the tightness of connections among nodes assigned to the same cluster within a network, with a range of [−1, 1]. A Q value closer to 1 indicates stronger internal connections within clusters and weaker inter-cluster connections, suggesting superior clustering performance. Typically, Q > 0.3 is regarded as indicative of a significant cluster structure. The S value evaluates the degree to which individual nodes align with their assigned clusters, ranging from [−1, 1]. An S > 0.5 implies high intra-cluster consistency, while S > 0.7 indicates highly reliable clustering results. The specific Q and S metrics obtained for our keyword clustering are presented in Section 3.

VOSviewer (version = 1.6.20) [35] was employed to conduct statistics on publication volume by country and inter-country collaboration networks. To ensure the reliability and interpretability of the visualizations, a full counting method was applied. Thresholds were meticulously selected to filter out isolated nodes. Specifically, the minimum number of documents for a country and an author to be included was set to 2. Additionally, for the author collaboration network, the minimum cluster size was set to 15 to focus on substantial and representative collaborative groups. The bibliometrix package (version = 4.3.0) in R was used for annual publication volume statistics, total citations by country, average citations per country, contribution of authors to publications, and contribution of journals to publications. Standard default parameters were applied for the descriptive bibliometric data extraction.

## 3. Results

### 3.1. Annual Productivity Trends Analysis

**Figure 1:**
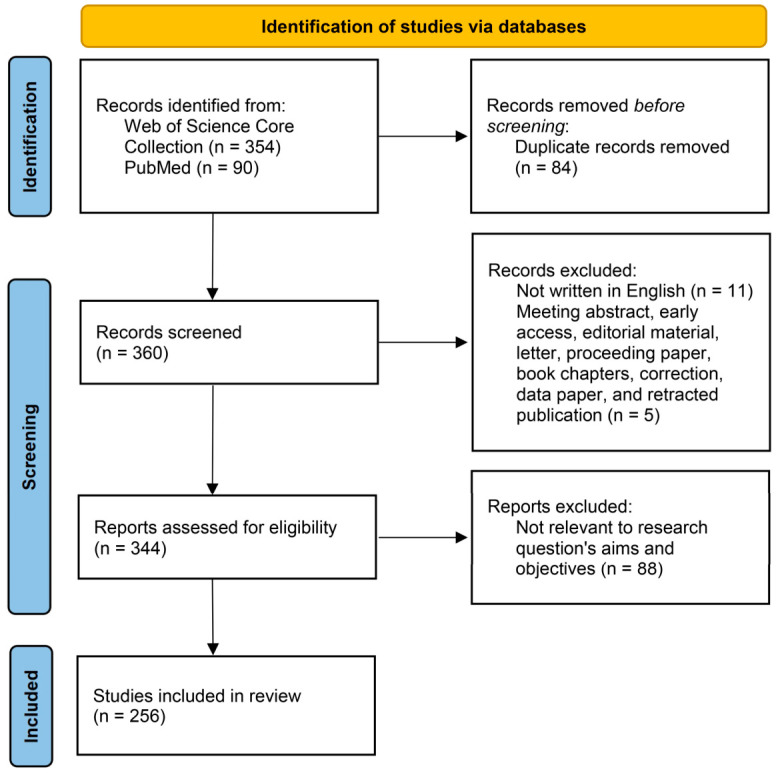
Flowchart of the search strategy and exclusion criteria.

The data utilized in this study were obtained from the WOSCC and PubMed databases. A systematic search conducted in these databases identified articles pertaining to nursing interventions for breast cancer patients undergoing radiotherapy. After the removal of duplicate records, a total of 360 articles remained. Following a rigorous selection procedure that excluded non-English literature and non-research papers or reviews, additional undesired literature was eliminated through full-text screening. As a result, a total of 256 articles, including 215 research articles and 41 reviews, were incorporated into the analysis (Figure 1).

**Figure 2:**
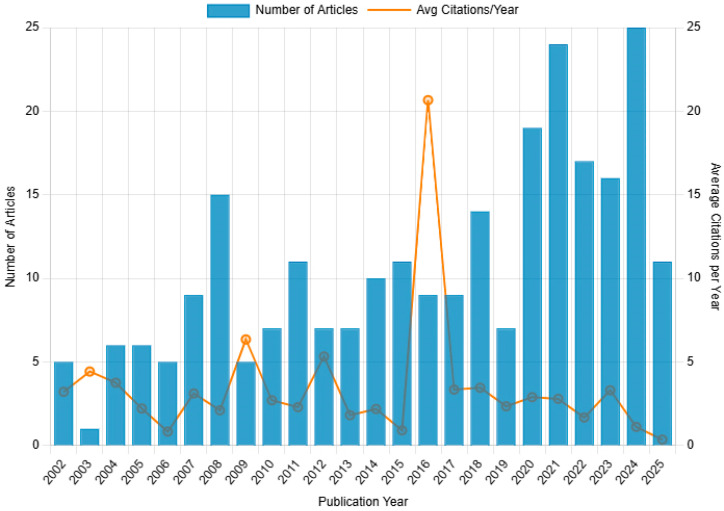
Temporal trends in publication output and citation impact of nursing intervention research for breast cancer radiotherapy patients (2002–2025).

The findings of the bibliometric analysis suggest that research on this topic demonstrates characteristics of temporal evolution. Regarding publication output, the annual number of publications fluctuated at a low level, varying from 1 to 9 articles between 2002 and 2007 (Figure 2). Although there was a transient peak of 15 articles in 2008, the publication count underwent moderate fluctuations, ranging from 5 to 14 articles per year from 2009 to 2019. The field entered a stage of rapid growth in 2020. It then maintained a considerably higher volume, with 16 to 25 articles annually between 2020 and 2024. This pattern reflects growing research interest in this domain. In terms of academic influence, the trend presents a marked contrast to that of publication output. Early literature (from 2002 to 2004, 2009, and 2012) exhibited relatively strong academic influence, with an average citation frequency ranging from 3.22 to 6.35 times per year. Notably, articles published in 2016 had an average citation frequency as high as 20.66 times, suggesting that this year may have made substantial scholarly contributions.

### 3.2. Contribution of Countries to Publications

**Figure 3:**
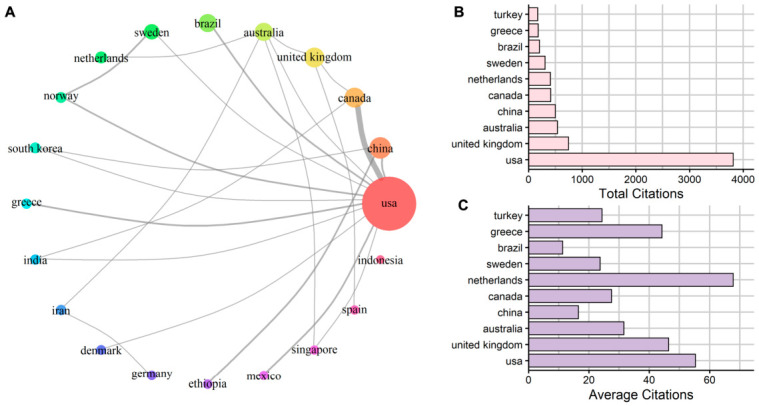
Contribution of country/region to publications. (**A**) The top 20 countries/regions by publication volume. The size of the circles represents publication volume, while the connecting lines in the center indicate the collaboration relationships between countries, with the thickness of the lines representing the number of collaborative papers. (**B**) The top 10 countries/regions by total citation count. (**C**) The top 10 countries/regions by average citation count per article.

Based on the geographical distribution analysis of corresponding authors, the global research output on this topic demonstrates significant disparities among major countries (Figure 3A). In terms of publication quantity, the United States (USA) leads with 82 articles, which is 3.3 times that of China (25 articles), the second-highest contributor. Subsequently, both Canada and the United Kingdom have 21 articles each. The international collaboration network is centered around the USA, which has established cooperative relationships with 12 of the top 20 countries ranked by publication output. Among these, the most frequent collaboration is with Canada (six co-publications), while the cooperation with other countries ranges from one to two instances.

An analysis was conducted on the citation impact of different countries in this field (Figure 3B). The United States takes the lead, with a total of 3813 citations and an average of 55.3 citations per article. The United Kingdom closely follows, having a total of 743 citations and an average of 46.4 citations per article. In contrast, despite the Netherlands having a relatively lower total citation count (407 citations), it shows the highest average citations per article, reaching 67.8 (Figure 3C). Australia and China rank third (537 citations) and fourth (496 citations), respectively, in terms of total citations. Nevertheless, China’s average citations per article are significantly lower, standing at 16.5. Canada, Sweden, Brazil, Greece, and Turkey also present commendable citation metrics.

### 3.3. Contribution of Institutions to Publications

**Figure 4:**
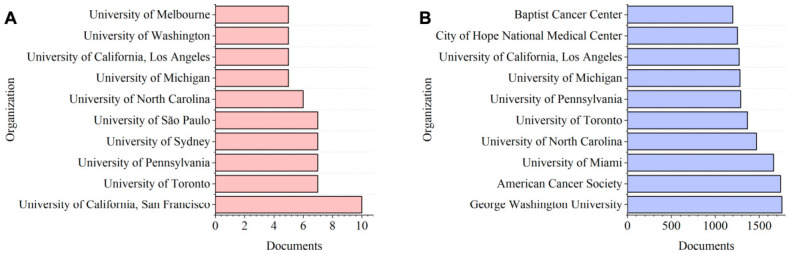
Top 10 institutions by publication volume and total citations. (**A**) Top 10 institutions by publication volume. (**B**) Top 10 institutions by total citations.

Based on bibliometric analysis, the findings concerning institutional productivity and impact are presented. In terms of publication quantity, the University of California, San Francisco was identified as the most productive institution, with 10 publications. It was succeeded by the University of Toronto, the University of Pennsylvania, the University of Sydney, and the University of São Paulo, each having 7 documents. Regarding citation influence, George Washington University and the American Cancer Society exhibited the highest impact, amassing 1759 and 1743 citations, respectively, from only 4 documents each (Figure 4). Several institutions, such as the University of Toronto, the University of Pennsylvania, the University of Michigan, the University of California, Los Angeles, and the University of North Carolina, ranked notably among both the top publishing institutions and the most-cited institutions. This indicates their substantial output and influence in this domain.

### 3.4. Contribution of Authors to Publications

**Figure 5:**
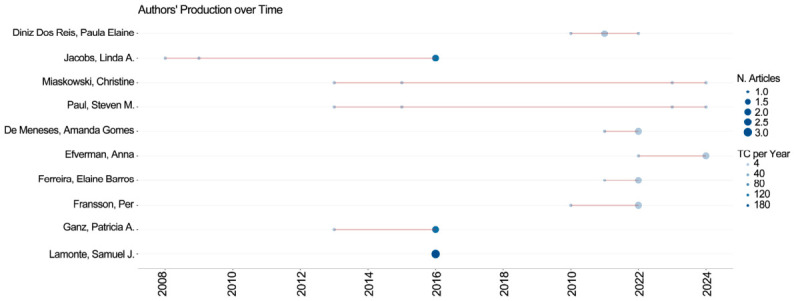
Contribution of authors to publications. A. The timeline analysis of the top 10 authors by publication volume. The x-axis represents the active publishing lifespan of the top authors within the studied field (from their first to their most recent publication). The size of the bubbles indicates the annual number of articles published (N. Articles). The color intensity of the bubbles represents the total citations per year (TC per Year).

An analysis of authors in this research domain reveals that the overall publication volume within the core author group is relatively limited. The four authors with the highest publication volumes, Linda A. Jacobs, Paula Elaine Diniz Dos Reis, Christine Miaskowski, and Steven M. Paul, have each published four papers (Table S1). A temporal analysis of the top 10 authors by publication volume (Figure 5) suggests that the active periods of these high-output authors differ. Among them, Christine Miaskowski and Steven M. Paul are among the authors with the longest active tenures in the field. Their publication records span from 2013 to 2024, which reflects their long-term academic involvement. Overall, this particular research area is lacking in authors with an extremely high number of publications.

### 3.5. Contribution of Journals to Publications

**Figure 6:**
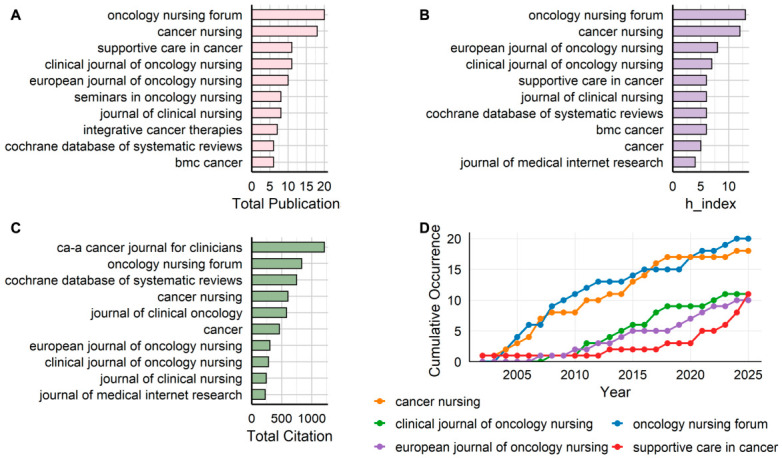
Contribution of journals to publications. (**A**) Total publications of the top 10 journals. (**B**) h-index of the top 10 journals. (**C**) Total citations of the top 10 journals. (**D**) Cumulative occurrence of selected journals from 2002 to 2025. In sub-figures (**A**–**C**), journals are listed on the y-axis with corresponding metric values on the x-axis. Sub-figure (**D**) is a line chart showing the cumulative occurrence of journals over time, with different colored lines representing different journals.

A bibliometric analysis of journals publishing research on this topic uncovered distinct patterns of publication output and influence. As depicted in Figure 6A, the *Oncology Nursing Forum* was recognized as the most prolific journal, contributing 20 articles, followed by *Cancer Nursing and Supportive Care in Cancer*. In terms of the h-index, which gauges both productivity and citation impact, the *Oncology Nursing Forum* also held the highest position (h-index = 13), indicating its sustained influence within the field. *Cancer Nursing and Supportive Care in Cancer* ranked next (Figure 6B). Moreover, with respect to journal impact factors (IF) and JCR categories, the *Cochrane Database of Systematic Reviews* maintained the highest impact factor (IF = 9.4) among the top 10 journals. It was followed by the *Journal of Clinical Nursing* (IF = 3.5). Both journals are in the Q1 category (Table S2).

Nevertheless, the journal with the highest total citation count was *CA-A Cancer Journal for Clinicians*. Despite a relatively low publication volume, it amassed over 1000 citations, highlighting the significant impact of the research it published (Figure 6C). Lastly, the analysis of temporal trends (Figure 6D) indicated a consistent rise in cumulative publications from 2005 to 2025 among five prominent journals, namely *Cancer Nursing*, *Oncology Nursing Forum*, *Supportive Care in Cancer*, *Clinical Journal of Oncology Nursing*, and *European Journal of Oncology Nursing*. This reflects the increasing academic focus on this research area.

### 3.6. Keyword Clusters Analysis

**Figure 7:**
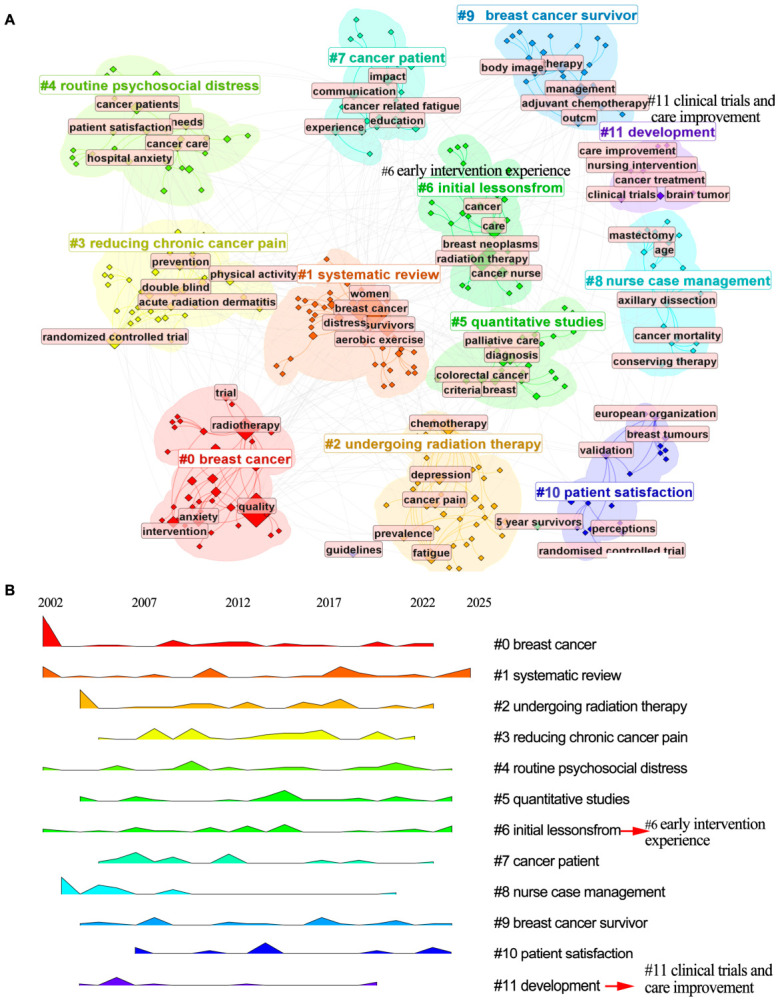
Keyword cluster analysis. (**A**) Keyword cluster network. Different colors represent different clusters, with the top 5 most frequent keywords displayed for each cluster. Node size reflects keyword frequency and inter-node linkages, indicating thematic associations. The text in the white matrix represents the theme of each cluster. (**B**) Keyword cluster time analysis. Each row represents a cluster, with the timeline from 2002 to 2025 depicted from left to right. The x-axis indicates the publication year, while the vertical peaks and the thickness of the colored bands reflect the annual frequency and research heat of each specific cluster theme. As indicated by the red arrows and text, the original algorithmic labels for cluster #6 (“initial lessonsfrom”) and cluster #11 (“development”) were manually refined to “early intervention experience” and “clinical trials and care improvement,” respectively, to more accurately reflect the actual nursing context of the clustered literature.

Keyword co-occurrence cluster analysis has identified twelve major research themes within this field from 2002 to 2025. The keyword clustering analysis resulted in a Modularity Q value of 0.5886 and a Mean Silhouette S value of 0.8807, suggesting that the cluster structure exhibits a high level of significance and the clustering results possess a high degree of reliability. As illustrated in the keyword cluster network (Figure 7A), the research topics are categorized as follows: #0 breast cancer, #1 systematic review, #2 undergoing radiation therapy, #3 reducing chronic cancer pain, #4 routine psychosocial distress, #5 quantitative studies, #6 initial lessonsfrom, #7 cancer patient, #8 nurse case management, #9 breast cancer survivor, #10 patient satisfaction, and #11 development. To guarantee accuracy and readability, certain automatically generated cluster labels were manually refined through an in-depth review of the underlying manuscript. The ambiguous label “#6 initial lessonsfrom” was revised to “#6 early intervention experience”. This cluster centers on early symptom tracking and proactive care models for early-stage breast cancer. For instance, it encompasses pilot studies reporting the preliminary experience of implementing nurse-enabled care models for early breast cancer [36]. It also includes clinical experiences in managing unplanned nursing interventions during the early stages of cancer treatment [37].

Moreover, “#11 development” was updated to “#11 clinical trials and care improvement”. The fundamental papers in this cluster mainly present randomized clinical trials and the design/development of novel nursing assessment tools targeting quality improvement. For example, studies in this cluster elaborate on the development and usability testing of mobile health applications for patients undergoing radiotherapy [38]. They also include randomized controlled trial protocols for digital health interventions featuring proactive nurse follow-up [39].

The keyword timeline analysis (Figure 7B) reveals the evolutionary trajectory of these research themes across a temporal dimension. Themes related to symptom management, such as #3 the mitigation of chronic cancer pain, and fundamental care components, such as #2 undergoing radiation therapy, demonstrated earlier research endeavors. In contrast, themes associated with the quality of life and integrated care models—including #9 breast cancer survivors, #4 routine psychosocial distress, and #8 nurse case management—have gained significant impetus or sustained consistent research efforts in more recent periods. This shift reflects an evolving research focus from the control of acute symptoms during radiotherapy towards long-term rehabilitation, psychosocial support, and systematic care management.

### 3.7. Keyword Evolution Analysis

**Figure 8:**
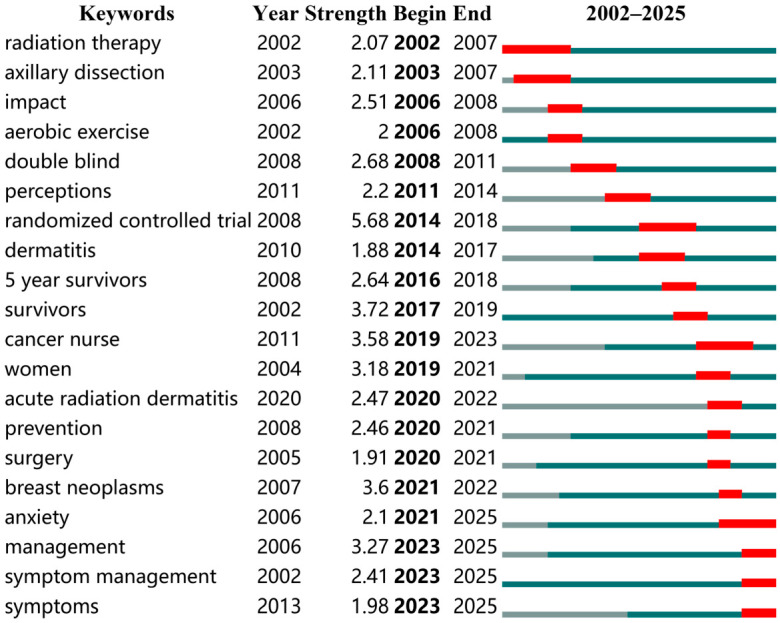
Top 20 keywords with the highest citation bursts. A “burst” indicates a sudden and significant increase in the occurrence frequency of a specific keyword over a defined period, reflecting the emerging trends and hot topics within the research field. Year: The year the keyword first appeared in the analyzed dataset. Strength: The statistical intensity of the burst. A higher strength value indicates a more dramatic surge in research interest for that topic. Begin/End: The starting and ending years of the burst period. Timeline (2002–2025): The timeline visualizes the lifespan and burst duration of each keyword. The red line segment indicates the exact duration of the burst (corresponding to the Begin and End years). The dark teal line represents the period during which the keyword was present in the literature but not experiencing a burst, while the light gray line indicates the time before the keyword’s burst or initial appearance.

Figure 8 depicts the top 20 keywords with the most substantial citation bursts from 2002 to 2025. The analysis uncovers distinct temporal patterns in the research focus. The keyword “randomized controlled trial” exhibited the highest burst strength (5.68) during the period from 2014 to 2018, followed by “survivors” (3.72, 2017–2019). Notably, keywords including “management” (3.27, 2023–2025), “symptom management” (2.41, 2023–2025), “anxiety” (2.10, 2021–2025), and “symptoms” (1.98, 2023–2025) display significant citation bursts. This suggests a shift in research emphasis towards comprehensive symptom management, especially psychological symptoms such as anxiety, and systematized nursing interventions. In contrast, earlier bursts were mainly related to treatment techniques and surgical approaches. Collectively, the burst data illustrate a transition from terms associated with initial treatment technologies to those centered on integrated care models and the management of physical and psychological symptoms.

## 4. Discussion

Although prior research has predominantly concentrated on the clinical impacts of specific nursing interventions and the function of nurses in radiotherapy care, quantitative bibliometric evidence within this area remains scarce. In this context, the current study offers a bibliometric overview of nursing interventions for breast cancer radiotherapy. By analyzing publications from 2002 to 2025, we delineate the knowledge framework and evolutionary trajectory of this field, thus furnishing a visual reference for future investigations.

### 4.1. Growth Trends in Research Output and the Importance of Breast Cancer Radiotherapy Nursing

This research indicates that the annual publication output in the domain of breast cancer radiotherapy nursing entered a phase of rapid growth commencing in 2020. The surge in citations in 2016 was mainly propelled by several landmark publications that redefined the field. Notably, the joint publication of the Breast Cancer Survivorship Care Guideline by the American Cancer Society and the American Society of Clinical Oncology in 2016 [40] offered comprehensive evidence-based recommendations for monitoring the late effects of radiotherapy, managing fatigue, and providing psychosocial support. This publication may have contributed to increased scholarly attention to this area. As an integral component of breast cancer treatment, especially for patients after breast-conserving surgery or those with high-risk factors after mastectomy, radiotherapy has been demonstrated to substantially reduce local recurrence rates and enhance survival outcomes [41,42,43]. With the development of precision medicine, treatment goals in breast cancer have gradually expanded beyond survival alone to include quality of life and psychosocial recovery. Consequently, nursing care has received growing attention in relation to radiotherapy-related problems such as radiation dermatitis, cancer-related fatigue, lymphedema, and psychological distress [40,44]. Progress in clinical practice may also have prompted further research aimed at addressing patients’ increasingly complex care needs.

### 4.2. Analysis of Academic Influence and Collaboration Patterns Across Countries and Institutions

Analysis at the country/region level indicates that the United States occupies a leading position in terms of publication volume, total citation count, and international collaboration networks, with especially robust cooperative links to countries such as Canada and Australia. This dense network among high-income nations implies a concentration of research resources and well-established infrastructures, which promotes the rapid dissemination of advanced nursing protocols within these regions. Notably, the Netherlands demonstrates the highest average citation rate per article, suggesting that its research may be centered on cutting-edge topics. In contrast, although China ranks second in publication output, its average citation rate is relatively low. Alongside these trends, the presence of Turkey among the top ten in citation metrics further underscores its increasing involvement in this field.

However, these observations should be interpreted with prudence due to the inherent bibliometric biases embedded in our methodology. At the institutional level, the findings are consistent with the national trends, indicating that high-productivity institutions are predominantly concentrated in North America. For example, the University of California, San Francisco, and the University of Pennsylvania in the United States, along with the University of Toronto in Canada, all rank among the top ten institutions in terms of publication output. While this reflects genuine research capacity, it is also magnified by the selection criteria of the databases used. Specifically, the WOSCC and PubMed tend to prioritize high-impact and multidisciplinary journals. As a result, nursing-focused literature indexed in other databases may be underrepresented. This methodological bias is empirically substantiated by our supplementary search. A subsequent pilot search conducted in the Cumulative Index to Nursing and Allied Health Literature (CINAHL) and Scopus using the identical core search string yielded 110 and 157 records, of which 65 were unique and not indexed in WOSCC or PubMed. Relative to our analyzed sample of 256 publications, this volume of uncaptured records suggests a significant indexation gap. Consequently, the observed geographic imbalance is not solely a manifestation of unequal research productivity but is also a methodological artifact. High citation counts associated with North American institutions may reflect the visibility of their publications within the WOSCC/PubMed ecosystem rather than superior global clinical impact. Therefore, the citation-based landscape may represent only part of the field. Although equitable international collaboration remains an important consideration [45], the current bibliometric map is constrained by database coverage and citation practices. It may therefore overlook regionally relevant care models published in lower-impact or non-indexed journals. Future studies using broader search strategies are needed to validate and extend these findings.

### 4.3. Evolution of Concrete Nursing Interventions and Clinical Relevance

A critical examination of keyword clusters, such as “#3 reducing chronic cancer pain” and “#4 routine psychosocial distress,” reveals that the scope and nature of clinical nursing care have undergone a substantial evolution. Rather than merely providing a descriptive account, our findings suggest a shift from isolated, symptom-centered care toward more structured and multidimensional care models.

One prominent trend pertains to the management of physical symptoms. The keyword cluster associated with chronic cancer pain and the recent emerging terms “symptom management” and “symptoms” indicate that symptom control has remained a core research focus, yet its scope has evolved over time. Earlier studies primarily addressed isolated acute toxicities with relatively straightforward supportive measures, such as topical skin care, routine patient education, and basic pain support. However, more recent research increasingly conceptualizes symptoms as co-occurring clusters that necessitate systematic assessment and coordinated intervention [46,47,48,49,50]. This shift is clinically significant because patients undergoing radiotherapy often experience multiple overlapping symptoms. Consequently, nursing care has transitioned from reactive management of individual symptoms to more structured approaches based on repeated assessment, personalized care planning, and timely adjustment of interventions. Standardized tools like the Edmonton Symptom Assessment System have demonstrated value in reducing the overall symptom burden, while digital symptom monitoring platforms represent an emerging strategy to support early detection and prompt nurse-led responses [51,52,53]. Nevertheless, despite their potential, such digital approaches may be more feasible in well-resourced settings, and their real-world impact on long-term outcomes and implementation burden still requires further evaluation.

A second major trend is the increasing emphasis on psychosocial and structured supportive care. The keyword cluster related to psychosocial distress and the emerging term “anxiety” indicate that emotional problems are increasingly recognized as core components of nursing care during radiotherapy rather than secondary concerns. This shift may be partly related to improved survival among patients with breast cancer, which has broadened clinical attention from acute treatment to longer-term well-being and survivorship. As survivorship extends, clinical attention has naturally expanded from acute-phase physiological treatment towards long-term psychological well-being and social reintegration. This shift is clinically significant because anxiety, depression, body image concerns, and fear of recurrence often coexist with physical symptoms and together deteriorate the quality of life [54]. This transition has been further reinforced by the emergence of clinical practice guidelines [40,55]. The publication of these guidelines has institutionalized the necessity of psychosocial support, spurring more extensive research to align with evolving clinical standards. Accordingly, nursing interventions have evolved from general emotional support and unstructured counseling towards more organized and evidence-based approaches, including psychoeducational programs, nurse-led case management, cognitive-behavioral strategies, mindfulness-based interventions, and navigation models [54,55,56,57]. These interventions suggest a broader nursing role across the treatment continuum, encompassing not only symptom relief but also emotional adaptation, self-management support, and survivorship care. At the same time, the literature indicates that their benefits may be more pronounced in selected patient subgroups than in unselected populations, highlighting the need for more targeted and standardized intervention pathways. Although supportive modalities are becoming more diverse, many studies still provide limited details on intervention components, duration, fidelity, and long-term sustainability. This lack of detail may hinder their translation into routine practice [58].

Collectively, these thematic changes suggest that the actual evolution of the field lies in three distinct shifts: from single symptoms to symptom clusters, from general supportive care to structured and theory-informed interventions, and from short-term toxicity management to survivorship-oriented quality-of-life support. These shifts are clinically relevant because they define where nursing practice makes its most distinctive contribution in breast cancer radiotherapy: not in replacing oncological treatment, but in optimizing the patient’s functional, emotional, and experiential outcomes during and after treatment.

### 4.4. Implications for Nursing Practice, Education, and Policy

The findings of this study have implications for understanding current priorities in nursing practice, education, and healthcare policy. In clinical practice, the observed shift in research focus from acute symptom management toward psychosocial support, symptom clusters, and case management suggests growing interest in more proactive and comprehensive approaches to patient care. These trends may provide a useful reference for clinical teams seeking to strengthen symptom screening, standardized assessment, psychosocial support, and interdisciplinary coordination. At the same time, further empirical studies are needed to determine how these approaches can be most effectively integrated into routine practice.

These trends also have implications for nursing education, highlighting the need for training that extends beyond technical radiotherapy care to include psychosocial screening, non-pharmacological approaches, and digital health management. From a policy perspective, healthcare institutions may consider supporting specialized roles to improve care continuity. Moreover, the geographic concentration of publications and collaborative activities indicates that persistent disparities remain in this field. Future research could further explore how nursing research models can be adapted to diverse cultural contexts and resource-constrained settings, while also promoting broader global collaboration. This is particularly important for strengthening the participation of low- and middle-income countries.

## 5. Limitations

This study has several limitations that should be considered when interpreting the findings.

Firstly, to ensure data consistency and the quality of indexed records, only English-language publication databases were incorporated. This language constraint may introduce selection bias, potentially leading to the oversight of relevant culturally specific or localized nursing interventions from non-English-speaking regions. Moreover, the search strategy utilized high-frequency general subject terms, failing to comprehensively integrate synonyms, specific pathological type terminologies, or detailed non-pharmacological intervention terms. As a result, this might have influenced the comprehensiveness of retrieving studies on particular subtypes and inadvertently excluded relevant non-pharmacological nursing interventions (e.g., telephone-based support), thereby restricting the scope of our analysis regarding specific nursing modalities.

Secondly, to uphold the rigor of peer review, non-primary documents such as conference abstracts, editorials, and book chapters were excluded. This may be a limitation because conference abstracts can reflect emerging findings before they appear in full-text publications. Thus, their exclusion may have caused us to miss some early-stage developments or alternative perspectives.

Thirdly, our analysis was confined to the WOSCC and PubMed. Although these are widely acknowledged as authoritative and high-quality sources for bibliometric analysis in the medical field, they may not encompass the entire spectrum of nursing literature. Specifically, databases like CINAHL are highly specialized in nursing and health services research, while Scopus offers extensive multidisciplinary indexing that may not be fully reflected in the WOSCC or PubMed. As specifically mentioned in Section 4.2, supplementary searches were conducted in CINAHL and Scopus, and 65 unique records were identified that were not indexed in the WOSCC or PubMed. In comparison to the analyzed sample of 256 publications, this volume of uncaptured records indicates that some nursing-specific publications might have been omitted, potentially affecting the comprehensiveness of our findings.

Fourthly, the relatively small sample size of 256 sources, which results from our strict inclusion criteria to ensure high quality, may limit the robustness and macroscopic generalizability of our network analyses when compared to larger bibliometric studies. However, it is essential to consider this number within the context of oncology nursing bibliometrics, where the volume of retrieved literature naturally varies according to the specific research scope. For example, recent bibliometric analyses on other oncology nursing fields have examined datasets of varying sizes, such as oncology genetic nursing (658 records) [59] and general liver cancer nursing (509 records) [60].

Fifthly, our database search was carried out in September 2025, creating a time gap of several months before manuscript submission. Given the rapid expansion and dynamic nature of the literature in this field, this temporal lag implies that the most recently published studies were not included, which may affect the timeliness and reliability of our latest findings.

Sixthly, recently published high-quality works often appear underrepresented in citation-based analyses simply because they have not had sufficient time to accumulate citations.

Finally, there are inherent limitations to bibliometric methodology. While bibliometrics offers valuable macroscopic insights into structural trends, it heavily relies on quantitative metrics and is ultimately unable to capture the nuanced content depth or the true scientific quality of the analyzed publications.

## 6. Conclusions

This bibliometric analysis indicates that research on breast cancer radiotherapy nursing has increased in recent years. While earlier studies predominantly focused on acute physical toxicities, a continuous paradigm shift is underway toward long-term psychosocial care and systematic case management. These findings have distinct implications for both clinical practice and research. Clinically, healthcare systems should consider shifting from isolated symptom control toward the integration of nurse-led, holistic interventions into standardized care pathways. In research, greater international collaboration may help address geographic disparities. This may be particularly relevant for low- and middle-income countries and may contribute to supporting long-term patient survivorship and quality of life.

## Data Availability

No new data were created or analyzed in this study. Data sharing is not applicable to this article.

## Supplementary Materials

The following supporting information can be downloaded at https://www.mdpi.com/article/10.3390/nursrep16070210/s1: Table S1: Top 10 most productive authors; Table S2: Top 10 most productive journals.

## Public Involvement Statement

No public involvement in any aspect of this research.

## Guidelines and Standards Statement

This manuscript was drafted against the Preliminary guideline for reporting bibliometric reviews of the biomedical literature (BIBLIO): a minimum requirements for review research.
